# Identifying the generalizable controls on insect associations of native and non‐native trees

**DOI:** 10.1002/ece3.11265

**Published:** 2024-05-12

**Authors:** Andrew V. Gougherty, Maartje Klapwijk, Andrew M. Liebhold, Angela Mech, Jiří Trombik, Songlin Fei

**Affiliations:** ^1^ USDA Forest Service Northern Research Station Delaware Ohio USA; ^2^ Department of Ecology Swedish University of Agricultural Sciences Uppsala Sweden; ^3^ USDA Forest Service Northern Research Station Morgantown West Virginia USA; ^4^ Faculty of Forestry and Wood Sciences Czech University of Life Sciences Prague Czech Republic; ^5^ School of Biology and Ecology University of Maine Orono Maine USA; ^6^ Department of Forestry and Natural Resources Purdue University West Lafayette Indiana USA

**Keywords:** evolutionary isolation, geographic ranges, insect–tree associations, invasive species, native trees, novel interactions, phylogeny

## Abstract

Trees growing outside their native geographic ranges often exhibit exceptional growth and survival due in part to the lack of co‐evolved natural enemies that may limit their spread and suppress population growth. While most non‐native trees tend to accumulate natural enemies over time, it remains uncertain which host and insect characteristics affect these novel associations and whether novel associations follow patterns of assembly similar to those of native hosts. Here, we used a dataset of insect–host tree associations in Europe to model which native insect species are paired with which native tree species, and then tested the model on its ability to predict which native insects are paired with which non‐native trees. We show that native and non‐native tree species closely related to known hosts are more likely to be hosts themselves, but that native host geographic range size, insect feeding guild, and sampling effort similarly affect insect associations. Our model had a strong ability to predict which insect species utilize non‐native trees as hosts, but evolutionarily isolated tree species posed the greatest challenge to the model. These results demonstrate that insect–host associations can be reliably predicted, regardless of whether insect and host trees have co‐evolved, and provide a framework for predicting future pest threats using a select number of easily attainable tree and insect characteristics.

## Graphical abstract

Trees growing outside their native geographic ranges often exhibit exceptional growth and survival due in part to the lack of co‐evolved natural enemies that may limit their spread and suppress population growth. Here, we used a dataset of insect–host tree associations in Europe to model which native insect species are paired with which native tree species, and then tested the model on native insects and non‐native trees. We show that native and non‐native tree species closely related to known hosts are more likely to be hosts themselves, but that native host geographic range size, insect feeding guild, and sampling effort similarly affect insect associations.

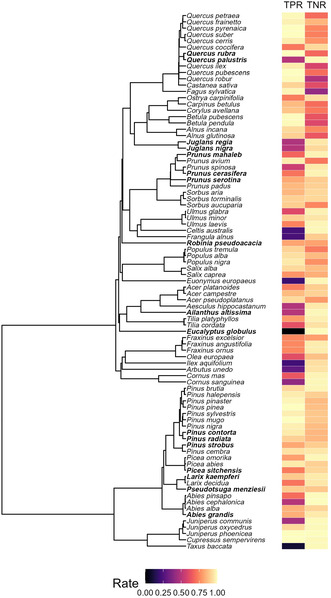

## 1 INTRODUCTION

The success of plants outside their native geographic ranges, whether introduced intentionally or accidentally, is affected by many factors, but one prevailing hypothesis is that the success of non‐native plants is due to the lack of natural enemies (i.e., enemy release) (Keane & Crawley, 2002), which would otherwise limit their spread and suppress population growth. The lack of natural enemies is often a desirable characteristic of cultivated plants (Lombardero et al., 2008), but can facilitate the ecological damage caused by some invasive plants (e.g., replacement of native species, reduction in soil fertility, shifts in water availability) (Castro‐Díez et al., 2021; Wolfe, 2002). While enemy release has frequently been observed in non‐native plants, many non‐native plants accumulate pests over time either through subsequent invasions by specialist herbivores or plant pathogens from the native range (e.g., Hurley et al., 2016; Medzihorský et al., 2023), or via native insects expanding their host breadth (Crous et al., 2017; Hawkes, 2007; Hurley et al., 2016). Native insects may be able to immediately utilize non‐native species or, over successive generations, may adapt to newly available hosts (Branco et al., 2015; Brändle et al., 2008; Siemann et al., 2006; Strong, 1974; Strong et al., 1977). These novel interactions between native insects and non‐native plants can be problematic for non‐native plants, as hosts may have no evolved resistance against novel insect herbivores and may be preferred compared to native hosts (Parker & Hay, 2005; Sunny et al., 2015). Furthermore, once an insect begins to utilize a non‐native host, the association provides a biological pathway for the insect to be transported to the native range of the host where it may devastate natural populations (e.g., Dang et al., 2022). Plants grown outside their native ranges, thus, have the potential not only to elucidate the mechanisms that facilitate insect–host interactions, but may also provide unique insights for identifying future pest threats that could impact non‐native plants in their native ranges (Eschen et al., 2019).

Despite the importance of insect enemies to the ecology and management of non‐native plants, it is unclear if the processes governing novel insect–host associations (that is, between native insects and non‐native hosts) are the same as those for native insect–host associations. If they are, it may be possible to predict novel associations, using host and insect characteristics from the native range, where insect and host ecologies may be better understood and documented. Numerous factors are likely to affect which insects feed on different plant species. First, if insects are limited to phylogenetically similar hosts, then the phylogenetic distance between native hosts and non‐native plants is likely important (Mech et al., 2019; Pearse & Altermatt, 2013; Schulz et al., 2021; Uden et al., 2022). The role of phylogeny is likely due to the phylogenetic pattern of traits (e.g., chemical or visual characteristics) used by insects to identify suitable hosts or coevolution of hosts and insects. While numerous studies have shown insect host breadth and damage are often phylogenetically clustered (Gilbert et al., 2012, 2015), this is not universal among insects, as some generalist insects are seemingly unaffected by host phylogeny, and can feed on a wide variety of distantly related hosts (Gougherty & Davies, 2022). In these cases, host breadth may be more limited by host availability and co‐occurrence, rather than relatedness. The effect of host phylogeny on insect associations is likely partially affected by the type of interaction between insects and hosts. Insects with specialized interactions, which require a tight coupling of host and insect physiologies, such as in gall‐forming insects, may be more phylogenetically restricted than other folivorous insects (Hardy & Cook, 2010) that feed on a wide array of species' leaves (Novotny et al., 2010).

Previous studies have also found that insect associations are affected by the geographic ranges of hosts (Branco et al., 2015; Joy & Crespi, 2012; Southwood & Kennedy, 1983). Plant species can be considered analogous to islands and may accumulate herbivores in the same way islands accumulate species, as expected from the theory of island biogeography—that is, species with large geographical ranges have greater apparency (Brändle & Brandl, 2001), which may increase the likelihood of new host associations forming (Janzen, 1968; Southwood & Kennedy, 1983). Large‐ranged host species may also cover larger climatic gradients or have higher local abundance (Sporbert et al., 2020), both of which could increase the opportunity of insect associations developing over time. Knowledge of insect–host associations could likewise be affected by the relative scientific knowledge of host species. Economically and ecologically important trees, for instance, those used for agriculture or forestry, are likely to be more closely monitored for pests that may impede their growth and productivity, compared to rare plants not considered ecologically or economically important.

Here, we sought to identify the principal drivers of native insect associations of native European trees and then test how well these relationships can predict novel associations between native insects and non‐native tree hosts. Predicting these novel associations can help guide the introduction and importation of new tree species for forestry and horticulture and can improve our understanding of enemy‐release and the accumulation of pest loads on non‐native plants. We hypothesize that insect–host associations will be strongly affected by host tree relatedness, as host breadth for many pests is phylogenetically conserved. We expect this to be particularly true for host specialists which may utilize only a small, phylogenetically‐circumscribed subset of available host species (Gilbert et al., 2012; Gougherty & Davies, 2022). We also expect that host geographic range size and the relative scientific knowledge of host trees will also play a positive role as large‐ranged, well‐studied species may accumulate more insect herbivores. Likewise, we hypothesize that, although non‐native plants may be inhabiting unique biotic and environmental conditions not present in their native range, the factors limiting insect associations are generalizable among native and non‐native plants, and thus the insect associations of non‐native plants can be reliably predicted.

## 2 METHODS

### 2.1 Data

A list of 85 native and non‐native tree species in Europe was compiled from the European Atlas of Forest Tree Species (San‐Miguel‐Ayanz et al., 2016). For each tree species, a list of insects known to utilize that species as a host was compiled from a variety of sources (see Table S1). These sources contain thousands of insect–host associations documented in the literature, but we acknowledge even these large databases may have some omissions. Thus, to ensure our data collection was standardized and reproducible, we limited our analysis to the insect–host associations characterized in these high‐quality, reliable databases. Each insect was researched to determine its taxonomic groupings, feeding guild (gall‐maker, folivore, reproductive plant feeder, sap‐feeder, or phloem/wood‐borer), and whether it was native to Europe or non‐native based on the Fauna Europea database (De Jong et al., 2014) and the International Non‐native Insect Establishment Database (Turner et al., 2021). Accuracy of the dataset was ensured through multiple steps that involved the removal of: (1) insect species not considered herbivores (e.g., parasitoids in Braconidae), (2) duplicated tree‐insect pairs due to spelling errors (e.g., *Alebra wahlbergi* vs. *A. wahlbergii*) or different Latin cases (e.g., *Phyllonorycter cerasicolella* vs. *P. cerasicolellus*), and (3) subspecies if the species was already listed for the tree species. In addition, insect species synonyms were identified, and subsequently removed, using the Global Biodiversity Information Facility taxonomic backbone accessed with the rgbif R package (Chamberlain & Boettiger, 2017). The final dataset included 1592 insect species.

### 2.2 Model predictors

We used seven predictors to model which native insects are associated with which host tree species. These included the mean phylogenetic distance between trees and known hosts of the insect, the variance of these distances, insect feeding guild, numerous host characteristics (geographic range size, and taxonomy), and sampling (representing the relative knowledge of host species).

Phylogenetic metrics were calculated using a phylogenetic tree created from V.PhyloMaker (Jin & Qian, 2019). Briefly, a megatree (Zanne et al., 2014) was pruned to the host species in our analysis, and any host trees not included in the megatree were included as a polytomy within the inclusive genus. Three phylogenetic metrics were calculated from this tree: mean distances to known host species, variance of distances to known hosts, and the minimum distance to the nearest native tree. The mean and variance distance were meant to capture how closely related trees are to known hosts of the insect, and how variable this was. In both cases, if a tree was a host, the zero distance was not included in the calculation. These two metrics were calculated for each insect–host pair included in the model. The minimum distance was included as a metric of how evolutionarily distinct tree species were with respect to the entire native tree community. This metric was calculated for each host tree.

Host characteristics included geographic range area and broad taxonomic grouping (i.e., angiosperm, gymnosperm). Species geographic range areas were based on the EU‐Forest database (Mauri et al., 2017), and represent the current distribution of tree species across the European Union and the United Kingdom, based on countries' national forest inventories. Because countries vary in the sampling density of their respective forest inventory plots, we drew a 10 km buffer around each plot containing a focal host tree and then merged the buffers. We then masked the merged buffer area with a map of forested areas based on the CORINE land cover database (European Environment Agency, 2023). Species range sizes were calculated from this masked buffer area.

Sampling effort was quantified as the approximate number of search results from GoogleScholar for each host species, reported on the first page of search results for the contemporary scientific name of the host species. Although this metric is not specific to the knowledge of insects of each host, it serves as a useful proxy for the relative amount of research that has been conducted on each tree species. Finally, we included broad host taxonomic grouping (angiosperm, gymnosperm) as a categorical variable in the model.

Insect feeding guild was included in the model as a categorical variable and included folivores (*n* insects = 803), gall‐formers (*n* = 135), reproductive‐feeders (*n* = 11), sap‐feeders (*n* = 411), and wood‐borers (including bark‐ and phloem‐feeders, *n* = 232). Insects found to belong to multiple guilds were consolidated to a single guild, most common to the species.

### 2.3 Model and predictions

In total, our model was trained on the occurrence of 1592 insects on 68 native hosts. The insect–host association model was fit as a mixed effect logistic regression model using the lme4 package (Bates et al., 2015), and was fit as:

$$\begin{array}{c} {\text{isHost}}_{i}∼\text{Binomial}\left(n=1,{\text{prob}}_{\text{isHost}=1}=\hat{P}\right),\\ \log\left[\frac{\hat{P}}{1-\hat{P}}\right]=\alpha_{j\left[i\right]}+\beta_{1}\left(\text{mean evol}.\;\text{dist}\right)+\beta_{2}\left(\mathrm{var}.\;\text{evol}.\;\text{dist}\right)+\beta_{3}\left({\text{group}}_{\text{Gymnosperms}}\right)\\ +\beta_{4}\left(\text{citations}\right)+\beta_{5}\left(\text{range size}\right)+\beta_{6}\left(\text{evol}.\;\text{dist}.\;\text{nearest native}\right),\\ \alpha_{j}∼N\left(\gamma_{0}^{\alpha }+\gamma_{1}^{\alpha }\left({\text{guild}}_{\text{Gall}}\right)+\gamma_{2}^{\alpha }\left({\text{guild}}_{\text{Reproductive}}\right)+\gamma_{3}^{\alpha }\left({\text{guild}}_{\mathrm{Sap}}\right)+\gamma_{4}^{\alpha }\left({\text{guild}}_{\text{Wood}}\right),\sigma_{\alpha_{j}}^{2}\right),\text{for insectSpecies}\;j=1,\ldots ,J, \end{array}$$

where *α* is the intercept, and *β* are beta coefficients, extracted with the equatiomatic package. The model was trained on native hosts and native insect associations and non‐associations (*n* = 108,256 total insect–host pairs), and tested on non‐native hosts and native insects (*n* = 27,064). Non‐native trees included 17 species with native geographic ranges outside of Europe, including *Abies grandis*, *Ailanthus altissima*, *Eucalyptus globulus*, *Juglans nigra*, *Juglans regia*, *Larix kaempferi*, *Picea sitchensis*, *Pinus contorta*, *Pinus radiata*, *Pinus strobus*, *Prunus cerasifera*, *Prunus mahaleb*, *Prunus serotina*, *Pseudotsuga menziesii*, *Quercus palustris*, *Quercus rubra*, and *Robinia pseudoacacia*.

All continuous variables were log‐transformed and scaled before inclusion in the model (mean of zero, standard deviation of 1.0). The maximum correlation between any two continuous variables used in model training was between host geographic range size and sampling effort (Pearson's correlation coefficient, *r* = .71), all other pairs of variables were weakly correlated (all |*r*| < .3). In addition to the predictor variables described above, each insect species was allowed to have a random intercept. Model performance was measured using *R* ^2^ (estimated with the sjPlot package; Lüdecke, 2023), area under the receiver operating curve (AUC) (using the pROC package; Robin et al., 2011), total accuracy, true‐positive rate (TPR), and true‐negative rates (TNR). Here, the true‐positive rate represents the proportion of insect hosts that are accurately predicted, while the true‐negative rate represents the proportion of non‐hosts that are accurately predicted. These metrics were calculated across the entire dataset, for training and testing sets separately, as well as for each host and insect separately, and for insect guilds in aggregate. For performance metrics that required a binary response, the continuous prediction was thresholded using the value that maximized AUC. To better understand which insect–host associations were best predicted, we tested for phylogenetic signal (Pagel's lambda) in host TPR and TNR, and their relation to host evolutionary distinctiveness. The full dataset used in model fitting is available at https://doi.org/10.5061/dryad.3n5tb2rrx 2024.

## 3 RESULTS

**FIGURE 1:**
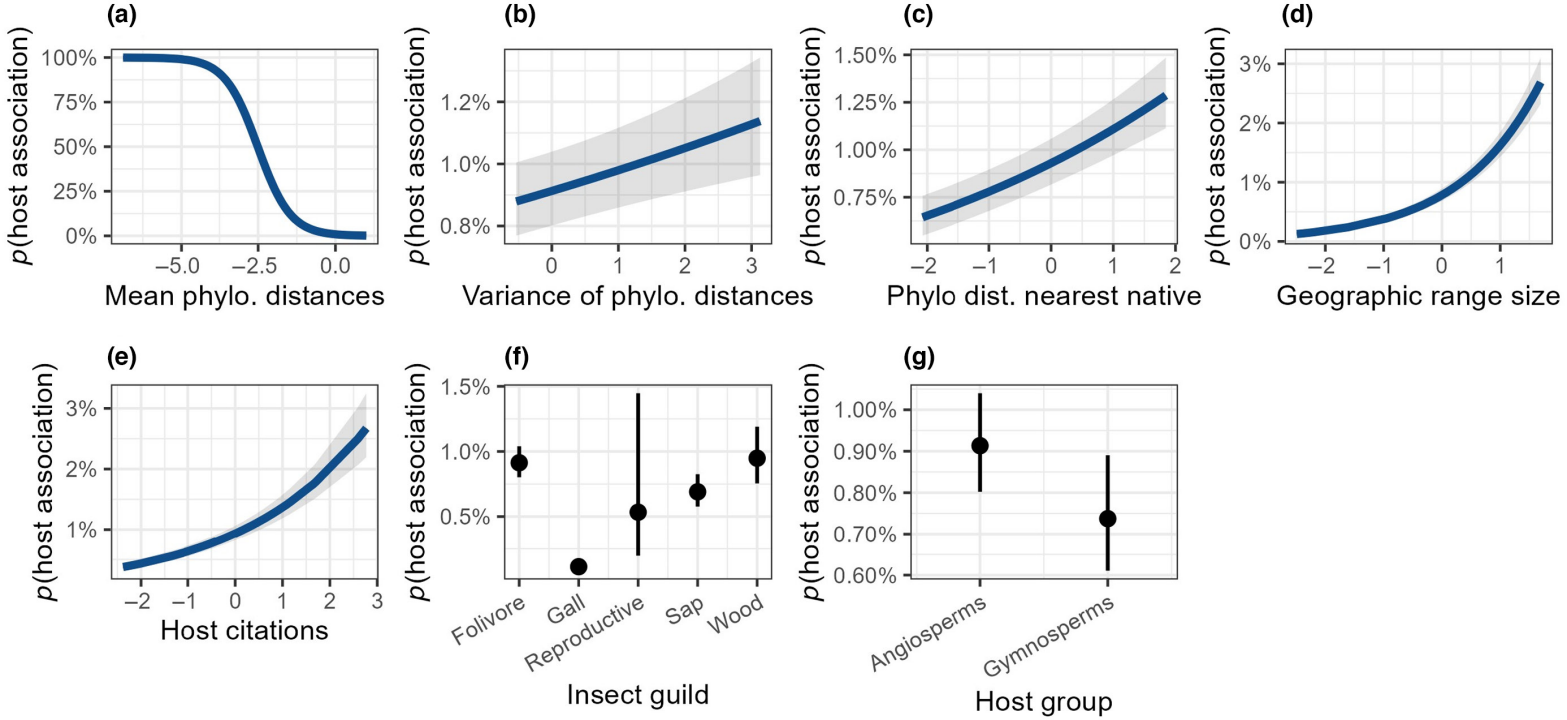
Marginal effects of predictors used in a mixed effect model of host–insect associations. Continuous variables were scaled and centered before inclusion in the model. The *y*‐axes show the predicted probability of host–insect association, indicated by *p*(). Note each panel has a unique *y*‐axis to ensure the shape and directionality of the variables was visible. The large *y*‐axis range for phylogenetic distance indicates its prominence in the model. Shaded areas in (a)–(e) and whiskers in (f) and (g) represent the 95% confidence interval. Each of the continuous variables was significantly associated with insect–host associations. For insect guilds, galling and sap‐feeding insects were significantly different from the reference guild (folivores), and gymnosperms were significantly different from angiosperms. See also Table 1.

In general, the model had good predictive ability (Table 2, Tables S2 and S3). The pseudo *R* ^2^ was .50, when considering only the fixed effects, but was .70 when the random effects of insect species were included. Insect–host associations were most strongly predicted by the mean evolutionary distance between trees and known hosts of the insect—consistent with most insects being phylogenetically restricted in their host breadth (Tables 1 and 2, Figure 1, Figure S1). The variance of distances was also important, and had a positive effect on associations, indicating that an association was more likely when hosts were both closely related to some hosts and distantly related to other hosts (Figure 1), potentially reflecting the wide host breadth of generalist herbivores, or a tendency of insect hosts to be clustered on multiple different parts of the host phylogeny. Geographic range size and sampling effort were both positively associated with the probability of insect associations, consistent with more widespread and intensively studied tree species having a greater possibility of insect associations (Table 1). Insect feeding guild was also important, with galling insects, in particular, tending to have fewer associations than insects in other guilds (Figure 1).

**TABLE 1:** Parameter estimates from the mixed model predicting insect–tree associations.

Predictors	Log‐odds	CI	*p*
(Intercept)	−4.84	−4.98 to −4.71	**<.001**
Mean evol. distance	−1.88	−1.92 to −1.84	**<.001**
Variance evol. distances	0.07	0.03 to 0.11	**<.001**
Guild [gall]	−2.11	−2.45 to −1.78	**<.001**
Guild [reproductive]	−0.54	−1.55 to 0.47	.294
Guild [sap]	−0.28	−0.48 to −0.08	**.006**
Guild [wood]	0.04	−0.21 to 0.28	.763
Host group [gymnosperms]	−0.22	−0.37 to −0.06	**.007**
Citations	0.39	0.33 to 0.44	**<.001**
Range size	0.74	0.67 to 0.81	**<.001**
Evol. dist. to nearest native tree	0.18	0.14 to 0.22	**<.001**
Random effects			
*σ* ^2^	3.29		
*τ*_00 insectSpecies_	2.27		
ICC	.41		
*N*_insectSpecies_	1592		
Observations	108,256		
Marginal *R* ^2^/Conditional *R* ^2^	.497/.702		

*Note*: In total 108,256 native insect–native tree associations and non‐associations were used to train the model, and 22,288 native insect–non‐native tree associations/non‐associations were used to test the model. Table summary was generated with the R package sjPlot (Lüdecke, 2023). Marginal *R* ^2^ accounts only for the fixed effects in the model, while the conditional *R* ^2^ accounts for fixed and random effects.

Bold values indicate a *p*‐value less than 0.05.

**TABLE 2:** Validation statistics from a mixed model predicting insect–tree associations. The model was trained on native insect–native tree associations and non‐associations and was tested on native insect–non‐native tree associations/non‐associations.

Statistic	Training data	Testing data
	Native host–insect	Novel host–insect
AUC	0.95	0.93
Total accuracy	0.86	0.91
True‐positive rate	0.87	0.76
True‐negative rate	0.86	0.91

Each accuracy metric (AUC, accuracy, TPR, TNR) was high for the training data (all >0.85, native tree hosts) and was only slightly lower for the testing data (all >0.75, non‐native tree hosts). TPR and TNR were similar for native hosts (0.87 and 0.86, respectively) while TPR was lower than TNR for non‐native hosts (0.76 and 0.91, respectively)—indicating the model had a slightly reduced ability to predict associations than non‐associations for non‐native hosts.

**FIGURE 2:**
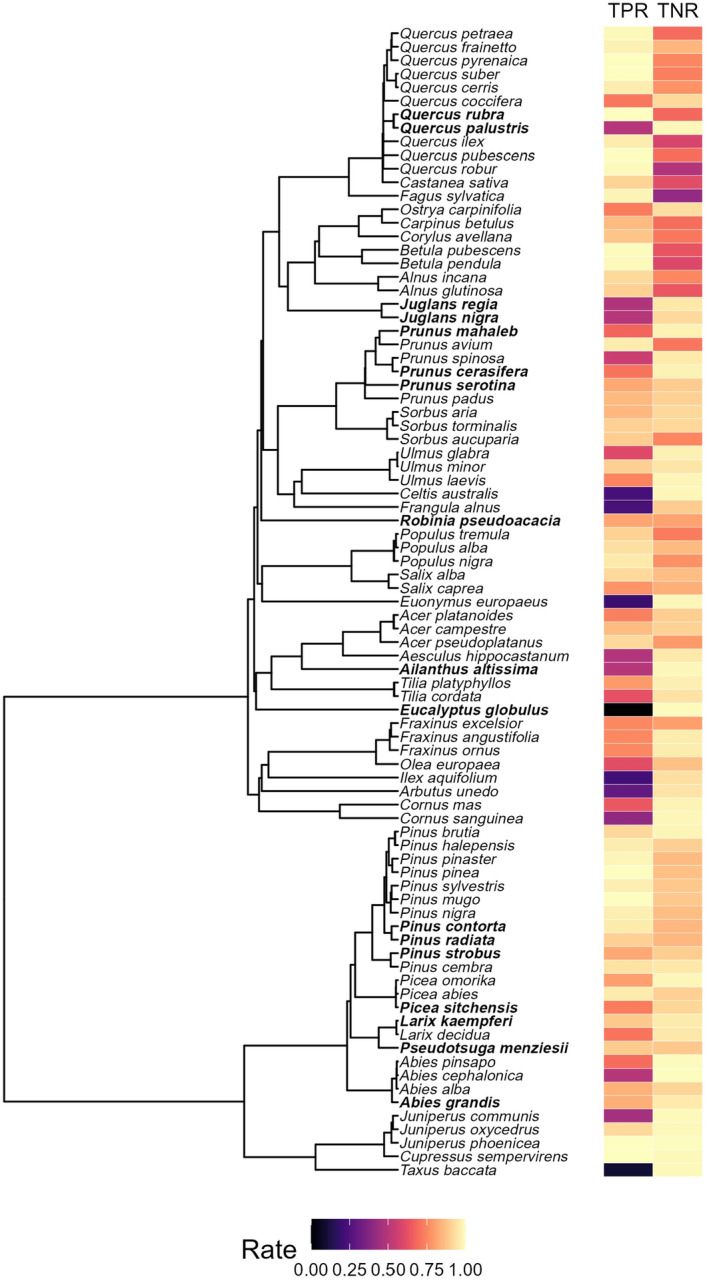
True‐positive rate (TPR) and true‐negative rate (TNR) rates for 85 host tree species included in our model. TPR indicates the proportion of insect associations that were accurately predicted by the model, and TNR indicates the proportion of non‐associations that were accurately predicted. For both TPR and TNR, higher values indicate greater predictive accuracy. Native species (*n* = 68) were used to train the model, while non‐native species (boldface, *n* = 17) were used to test the model. Visualization was made with ggtree (Yu et al., 2017).

Predictive ability also tended to be high for individual host species, but there was considerable variability (Figure 2). On average (±SD), TPR and TNR were 0.77 (±0.24) and 0.87 (±0.13), respectively, across host species, with ranges of 0–1.0 and 0.41–1.0. Species with numerous close relatives (e.g., *Quercus*, *Pinus*) often had high TPR, likely benefiting from a strong phylogenetic signal in many insects' host breadths. Consistent with this finding, both TPR and TNR had phylogenetic signals, indicating that closely related species tended to have similar rates. Non‐pine conifers tended to have among the highest TNR, in particular, *Abies* and *Juniperus*.

**FIGURE 3:**
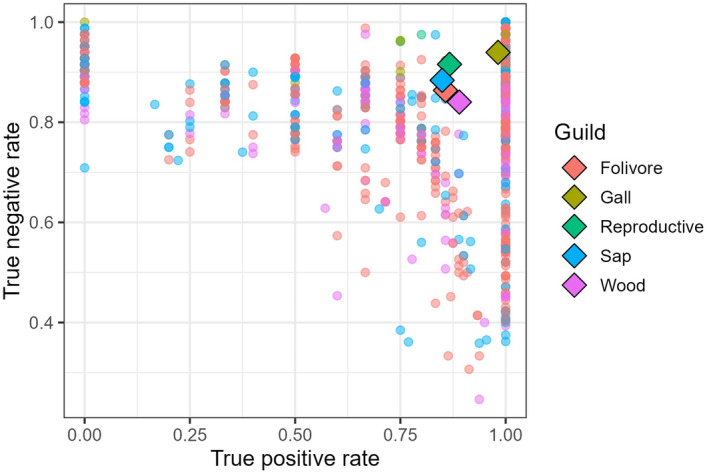
True‐positive rate (TPR) and true‐negative rate (TNR) of 1592 insect species used in our model. TPR indicates the proportion of hosts that were accurately predicted by the model, and TNR indicates the proportion of non‐hosts that were accurately predicted. Diamond symbols represent the aggregate TPR and TNR of five feeding guilds. For both TPR and TNR, higher values indicate greater predictive accuracy.

Values of TPR and TNR were also often high for individual insect species, but there was substantial variability (Figure 3). In aggregate, galling insects had the highest TPR and TNR, likely due in part to their small, phylogenetically circumscribed host breadths and intimate association with host physiology. TPR was lowest among insects that feed on sap (0.85), and TNR was lowest among wood borers (0.84), but both rates were high nonetheless.

## 4 DISCUSSION

Our work demonstrates that insect–host associations can be accurately predicted using a select number of relatively easily attainable insect and host traits. Like other studies (Gilbert et al., 2012; Pearse & Altermatt, 2013), we found that host phylogeny plays an important role in determining which insects were documented with which hosts, but we also found that host geographic range size, sampling, and insect feeding guild each play important roles. This work has important implications for identifying future, and currently undocumented, insect–host associations that may damage hosts in their non‐native ranges, and have potential to threaten native host populations if introduced abroad.

### 4.1 Phylogeny

Our results support previous findings that host phylogeny plays a crucial role in defining insect host breadths (e.g., Gilbert et al., 2012; Ness et al., 2011; Pearse & Altermatt, 2013), and implies that non‐native trees distantly related to the native tree community are less likely to be attacked by insect enemies—consistent with a greater opportunity for enemy release. Many non‐native plants will accumulate insect enemies over time (Branco et al., 2015; Brändle et al., 2008), and for agricultural and forestry species, the period of enemy release is often seen as an advantage to non‐native plants, as it may translate to increased growth and vigor, and less need for costly management to control insect enemies (Pearse & Rosenheim, 2020). The success of evolutionarily distinct species is readily seen in the wide planting (and success) of *Pinus* and *Eucalyptus* around the globe outside their native ranges (Wingfield et al., 2015), and taxonomically distinct non‐native plants generally (Pearse & Altermatt, 2013).

Despite the overall importance of host phylogeny in predicting host–insect associations, our analyses revealed considerable variability in individual insects' responses to phylogenetic distances. While many insects showed a strong response to host phylogeny, others appeared largely unaffected by phylogeny. Numerous insects (e.g., *Aphis fabae*, *Coccus hesperidum*, etc.), for instance, were recorded to utilize both gymnosperms and angiosperms, which diverged ~300 million years ago, and differ greatly in numerous functional traits (Stahl et al., 2013; Yang et al., 2022). Curiously, the insects recorded on angiosperms and gymnosperms, and other distantly related taxa, were not exclusively generalists (i.e., those that utilize many host species). Indeed, numerous insects were found to utilize only a few, distantly related species. The use of distantly related species, when congeners are available, is difficult to explain as one would expect insects to preferentially utilize trees similar to the hosts they are adapted to. This inconsistency, however, highlights the complexity of insect–host associations, and may be indicative of the importance of other factors on potential associations, including host geographic co‐occurrence, insect feeding behavior and/or host shifts following non‐native plant introduction (Agosta, 2006; Forister et al., 2015).

### 4.2 Geography and sampling

While several other predictive frameworks have been developed centered around using host phylogeny to predict insect (and pathogen) associations (Gilbert et al., 2012; Pearse & Altermatt, 2013), our results show that other factors are also important in predicting novel insect associations. For instance, we found that the size of the host geographic range had a strong positive effect on identifying insect associations. The tendency for tree species with large geographic ranges to be associated with more species‐rich communities of insect herbivores has been well documented and is considered to result in part from greater colonization probabilities, much like in island biogeography where large islands host more species‐rich communities (Brändle & Brandl, 2001; Southwood & Kennedy, 1983). Large‐ranged host species not only extend over larger spatial extents, but also may occur over larger climatic gradients and may have higher local abundance (Sporbert et al., 2020), each of which offers greater opportunity for insect and host distributions to overlap. The tendency for large‐ranged hosts to have a greater number of pest associations has been found in a variety of plant systems (Branco et al., 2015; Clay, 1995; Miller, 2012), and could provide an explanation for why non‐native hosts accumulate pests over time. As non‐native plants spread (or are introduced) to new regions and encounter the local pest community, pre‐adapted pests may shift to the novel host, especially if the host lacks any evolved resistance. Although native hosts are frequently preferred over novel, non‐native hosts, novel hosts are rarely “ecological traps” (Yoon & Read, 2016)—facilitating host breadth expansion and insect accumulation on novel hosts.

Sampling, as approximated by the number of citations attributable to host species, also played an important role on whether insects were observed as associations or non‐associations. This sampling effect suggests that some of the tree species that are not recorded as hosts in our data may in fact be hosts of certain insect species but those relationships have not yet been observed. This pattern is reflected in the false‐positive rate generated from the model predictions—that is, the insect–host pairs that the model predicts as associations, but are not actually observed in our data. These predictions may not necessarily be incorrect, as they may represent associations that are simply undocumented, or have not yet had the chance to occur (e.g., because host and insect geographic ranges do not overlap). Not surprisingly, several of the host species with the highest false‐positive rate are large ranged, well studied, and have multiple closely related species in Europe (e.g., *Fagus sylvatica*, *Quercus robur*), each of which increase the predicted probability of insect associations. Although more work would need to be done to establish whether these associations could occur in natural settings, they nevertheless suggest that these species may be target species for detecting future host jumps, and may be of particular concern as insects and hosts shift their ranges with contemporary and future climate change.

Perhaps more worrisome than the false positives were the observed host associations that were not predicted by the model. Not surprisingly, the false‐negative rate tended to be highest in monotypic taxa, likely due to a combination of the importance of phylogeny in the model but the lack of close relatives. Consistent with Darwin's naturalization conundrum, non‐native species were overrepresented among the monotypic taxa in Europe (e.g., *R. pseudoacacia*, *A. altissima*, *E. globulus*), although it is worth noting there are also numerous native monospecific genera included in our dataset (e.g., *Celtis australis*, *Euonymus europaeus*, *Aesculus hippocastanum*). The influence of phylogeny likely was not as important for species in monospecific genera, as they have no close relatives. These species likely benefited from the inclusion of geographic range size, insect feeding guild, and sampling in the model, but they perhaps require the most attention to fully understand their host associations.

### 4.3 Implications

Predicting native and novel insect associations has important implications for managing pests and predicting and planning for future insect invasions. While insects utilizing non‐native plants do not present an inherent risk to native populations, they can nevertheless indicate a threat if the insects are introduced to the native range. In one well‐known example, retrospective analyses revealed that American ash trees planted in China were susceptible to emerald ash borer (EAB), and this was known decades before EAB's introduction to the United States (Dang et al., 2022). While EAB is one high‐profile example, the propensity for tree pests to first attack trees outside their native ranges is not necessarily unique. In a global analysis, Gougherty and Davies (2022) showed that approximately ~23% of tree pests were known to occur only outside the native ranges of their tree host species—indicating many regions have native hosts for (potentially) hundreds of additional pests that are not yet known to occur. Identifying and predicting the actual insect–host associations before the associations can occur in the native range of the host can be an important tool to guide risk analyses to inform prevention and surveillance activities. Such predictions could be accomplished in the future through a combination of modeling efforts, like that described here, as well as by experimental sentinel plantings (Eschen et al., 2019; Raffa et al., 2023).

Predicting host–insect associations can also be useful for managing non‐native plants, whether seeking to reduce their ecological impacts or facilitate their growth. Using our approach to predict potential novel host associations, for instance, could be useful for identifying potential candidate agents for use in biocontrol and identifying which, and how many, non‐target plants may be at risk from spillover. While the effects of biocontrol agents on non‐target plants have historically been small (Suckling & Sforza, 2014), predicting host associations could potentially help guide experimentation to ensure native plant communities are not at risk from biocontrol agents. On the other hand, predicting host–insect associations could also help guide where non‐native plants, cultivated for agriculture or forestry, may best be planted to encourage enemy release and prevent pest damage. Phylogenetically distinct plants may need the least intervention against herbivore damage (Pearse & Rosenheim, 2020), especially if grown in relatively small areas, as opposed to expansive monoculture. Considering the regional plant and pest communities, thus can help to reduce the risk of unintended consequences when plants are introduced into new areas.

## AUTHOR CONTRIBUTIONS

**Andrew V. Gougherty:** Conceptualization (lead); formal analysis (lead); methodology (lead); writing – original draft (lead); writing – review and editing (lead). **Maartje Klapwijk:** Data curation (equal); methodology (equal); writing – review and editing (equal). **Andrew M. Liebhold:** Conceptualization (equal); methodology (equal); writing – review and editing (equal). **Angela Mech:** Data curation (equal); methodology (equal); writing – review and editing (equal). **Jiří Trombik:** Data curation (equal); methodology (equal); writing – review and editing (equal). **Songlin Fei:** Conceptualization (equal); methodology (equal); writing – review and editing (equal).

## Supporting information


Appendix S1

## Data Availability

Data used for model fitting are available at https://doi.org/10.5061/dryad.3n5tb2rrx.
