# Supplementary material for: Identifying the generalizable controls on insect associations of native and non‐native trees
Source: Ecol Evol. 2024 May 12;14(5):e11265. doi: 10.1002/ece3.11265 (PMC11089089; doi:10.1002/ece3.11265)
Supplement: Supplementary file 1 — Appendix S1 [file ECE3-14-e11265-s001.docx]

**Supplemental Material**

**Table S1.** Sources of insect-host records.

| Source | Reference |
| --- | --- |
| Aphids of the world database | Blackman 2006 |
| HOSTS - database of the world's Lepidopteran hostplants | Robinson 2010 |
| Insects and diseases damaging trees and shrubs of Europe | Kunca et al. 2013 |
| EPPO global database | EPPO 2023 |
| Artdatabanken | Swedish Agricultural University 2022 |
| Host Plants of World *Agrilus* | Jendek and Poláková, 2014 |
| SCALENET | Morales et al. 2016 |
| Discover Life | Pickering 2011 |
| The Database of British Insects and their Foodplants | Smith & Roy 2008 |

**References**

Blackman and Eastop (1994), EPPO (2021), García et al. (2016), Jendek and Poláková (2014), Kunca et al. (2013), Liljeblad (2021), Pickering (2011), Robinson et al. (2010), Smith and Roy (2008).

Blackman, R. L., & Eastop, V. F. (1994). Aphids on the World’s Trees: an Identification and Information Guide. New York: John Wiley & Sons.

EPPO (2021). European and Mediterranean Plant Protection Organization (EPPO) Global Database (available online). https://gd.eppo.int

García Morales M., Denno B. D., Miller D. R., Miller G. L., Ben-Dov, Y., Hardy N. B. (2016). ScaleNet: a literature-based model of scale insect biology and systematics. http://scalenet.info.

Jendek, E., & Poláková, J. (2014). Host plants of the world *Agrilus* (Coleoptera, Buprestidae): A critical review. Springer. 706 pp.

Kunca, A., Csoka, G., & Zubril, M. (2013). Insects and diseases damaging trees and shrubs of Europe. N. A. P. Édition. 535 pp.

Liljeblad, J. (2021). Swedish species observation system. https://www.artdatabanken.se/, accessed: 2021.

Pickering, J. (2011). Discover Life. http://www.discoverlife.org, accessed: MAY 2021.

Robinson, G. S., Ackery, P. R., Kitching, I. J., Baccaloni, G. W., & Hernández, L. M. (2010). HOSTS – a database of the world’s Lepidopteran hostplants. Natural History Museum, London. http://www.nhm.ac.uk/hosts, accessed: 2021.

Smith, R. M., and D. B. Roy. (2008). Revealing the Foundations of Biodiversity: The Database of British Insects and their Foodplants. *British Wildlife* 20: 17–25. http://nora.nerc.ac.uk/id/eprint/6995.

**Table S2.** Confusion matrix for model predictions of native host tree-insect associations. Ones represent known tree-insect associations and zeros represent non-associations. Values are row-standardized and thus represent the relative ability of the model to predict observed associations and non-associations. Values in parentheses are the total number of samples included in each category.

|  |  | Predicted | |
| --- | --- | --- | --- |
|  |  | 0 | 1 |
| Observed | 0 | 0.86 (88,145) | 0.14 (14,123) |
|  | 1 | 0.13 (753) | 0.87 (5,235) |

**Table S3.** Confusion matrix for model predictions of non-native host tree-insect associations. Ones represent known tree-insect associations and zeros represent non-associations. Values are row-standardized and thus represent the relative ability of the model to predict observed associations and non-associations. Values in parentheses are the total number of samples included in each category.

|  |  | Predicted | |
| --- | --- | --- | --- |
|  |  | 0 | 1 |
| Observed | 0 | 0.91 (24,397) | 0.09 (2,346) |
|  | 1 | 0.24 (77) | 0.76 (244) |


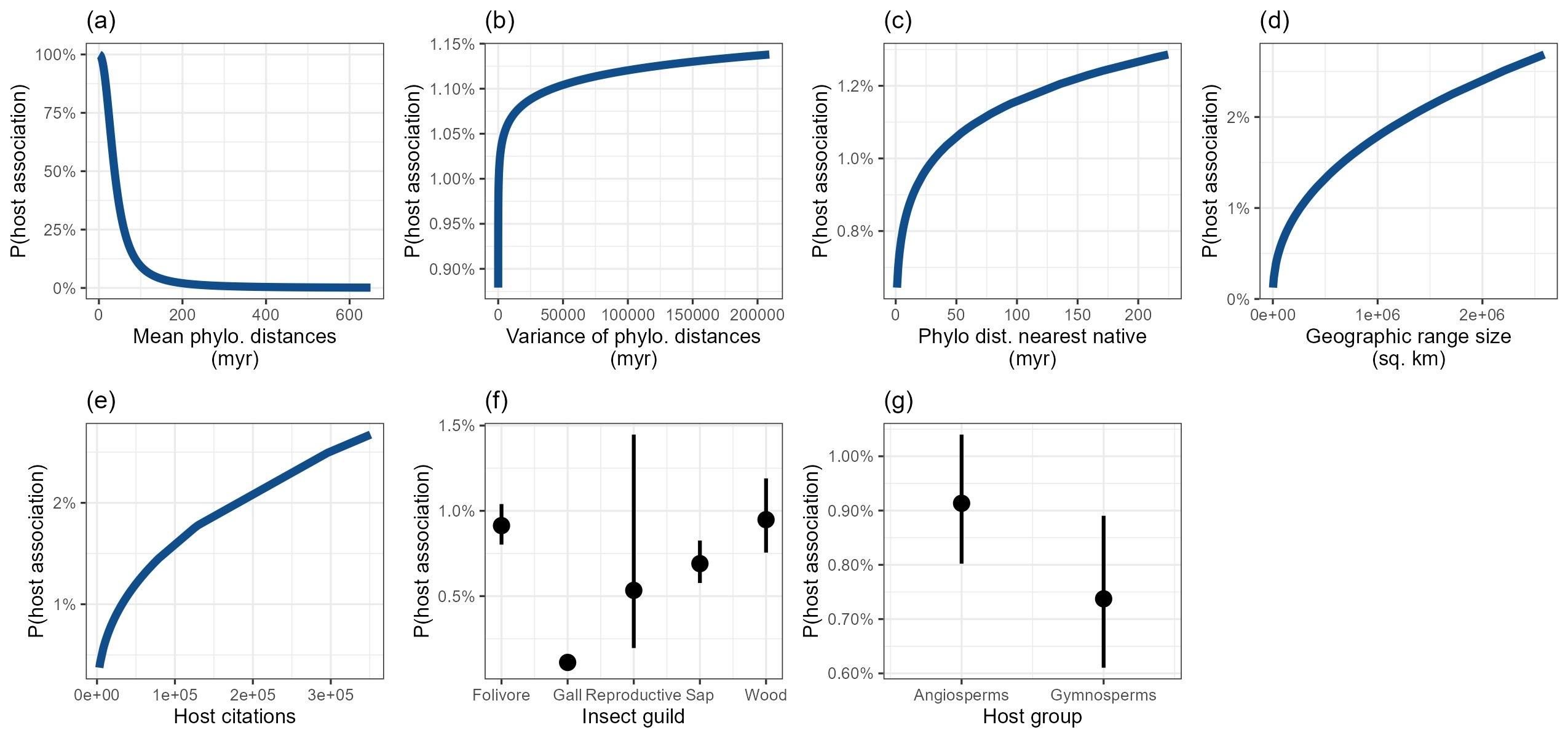


**Fig. S1.** Marginal effects of predictors used in a mixed effect model of host-insect associations. This shows the same information as to Fig. 1, but x-axes of the continuous variables have been back-transformed to the original scale. The y-axes show the predicted probability of host-insect association, indicated by P(). Note each panel has a unique y-axis to ensure the shape and directionality of the variables were visible. The large y-axis range for phylogenetic distance indicates its prominence in the model. Each of the continuous variables were significantly associated with insect-host associations. For insect guilds, galling and sap-feeding insects were significantly different from the reference guild (folivores), and gymnosperms were significantly different from angiosperms. See also Table 1.
